# The Cysteine Protease α-Clostripain is Not Essential for the Pathogenesis of *Clostridium perfringens*-Mediated Myonecrosis

**DOI:** 10.1371/journal.pone.0022762

**Published:** 2011-07-29

**Authors:** Anjana Chakravorty, Milena M. Awad, Thomas J. Hiscox, Jackie K. Cheung, Glen P. Carter, Jocelyn M. Choo, Dena Lyras, Julian I. Rood

**Affiliations:** Department of Microbiology, Monash University, Clayton, Australia; Oregon State University, United States of America

## Abstract

*Clostridium perfringens* is the causative agent of clostridial myonecrosis or gas gangrene and produces many different extracellular toxins and enzymes, including the cysteine protease α-clostripain. Mutation of the α-clostripain structural gene, *ccp,* alters the turnover of secreted extracellular proteins in *C. perfringens,* but the role of α-clostripain in disease pathogenesis is not known. We insertionally inactivated the *ccp* gene *C. perfringens* strain 13 using TargeTron technology, constructing a strain that was no longer proteolytic on skim milk agar. Quantitative protease assays confirmed the absence of extracellular protease activity, which was restored by complementation with the wild-type *ccp* gene. The role of α-clostripain in virulence was assessed by analysing the isogenic wild-type, mutant and complemented strains in a mouse myonecrosis model. The results showed that although α-clostripain was the major extracellular protease, mutation of the *ccp* gene did not alter either the progression or the development of disease. These results do not rule out the possibility that this extracellular enzyme may still have a role in the early stages of the disease process.

## Introduction

*Clostridium perfringens* type A is a Gram positive, spore forming anaerobic bacterium and is the causative agent of several human diseases, including gas gangrene or clostridial myonecrosis [1]–[5]. This syndrome is characterised by rapid tissue necrosis, a paucity of polymorphonucleocytes (PMNs) in the infected tissues and vascular leukostasis [1], [4], [6], [7]. *C. perfringens* type A produces two major extracellular toxins, α-toxin, which is essential for disease [1], and perfringolysin O, which has been shown to function synergistically with α-toxin to mediate disease progression [6], [7].

The extracellular cysteine protease α-clostripain was first discovered in *Clostridium histolyticum* [8]–[10] and a homologue was later identified in *C. perfringens* [11]–[14]. Both the *C. perfringens* and *C. histolyticum* α-clostripain proteins exist as heterodimeric polypeptides, consisting of a heavy chain and a light chain, which are held together by strong, non-covalent forces [8], [9], [15]. They are encoded by a single gene that contains a region encoding a nonapeptide linker [15], the polypeptide precursor is cleaved after secretion. Functionally, α-clostripains are arginine-specific endopeptidases that require calcium and reducing conditions for optimal *in vitro* activity [12], [13], [16]. They are classified as members of the C11 peptidase superfamily, which also includes gingipains and legumains [12], and are grouped based on their structural and functional similarity rather than their sequence similarity.

Other members of the C11 peptidase family include the gingipains HrgpA and RgpB from *Porphyromonas gingivalis.* These cysteine proteases play key roles during the infectious process [17]–[20]. They cleave important components of the innate immune system, thereby activating receptors that allow platelet aggregation [20], and cleave receptors on oral epithelial cells [19]. They also inactivate TNF-α and facilitate immune evasion [18] as well as disrupting the host cytokine response, inactivating IL-6 [17], [21]. Similarly, the cysteine protease SpeB from *Streptococcus pyogenes* has been shown to be important for disease and can inhibit immunoglobulin-mediated opsonisation and phagocytosis [22], [23] and can cleave and degrade human fibronection, vitronectin, and the C3 component of the complement system [24].

The role of *C. perfringens* α-clostripain in disease is not known. Previous workers [25] have made a single crossover mutation in the *C. perfringens* strain 13 α-clostripain gene, which has been designated as *ccp* [14] or *clp* [25]. Their results provided evidence that α-clostripain was required for the processing of secreted proteins since disruption of the *ccp* gene led to an increase in the levels of extracellular proteins [25]. In addition, α-clostripain production is positively and directly regulated by the VirSR two-component signal transduction system [14], [26], which also regulates perfringolysin O, α-toxin and collagenase production in *C. perfringens*. Recent *in vivo* studies have shown that when injected into the dorsal skin of mice, purified α-clostripain increases intravascular permeability in a dose-dependent manner [16], suggesting that α-clostripain may be responsible for the tissue swelling observed in clostridial myonecrosis [16]. In summary, it has been postulated that α-clostripain has the potential to affect the levels of active extracellular toxins and enzymes in the region surrounding *C. perfringens* cells and may therefore affect disease progression and virulence [13].

The objective of this study was to determine if α-clostripain was essential for disease. Accordingly, the *ccp* gene was insertionally inactivated, the mutation complemented with the wild-type *ccp* gene and the resultant panel of isogenic strains analysed for total protease activity, extracellular toxin production and virulence in the mouse myonecrosis model. The results showed that although α-clostripain is the major protease produced by *C. perfringens* it is not essential for disease.

## Results

### α-clostripain is the major protease produced by *C. perfringens*

To assess the role of α-clostripain in the pathogenesis of *C. perfringens*-mediated myonecrosis, the α-clostripain structural gene, *ccp*, in the strain 13 derivative, JIR325, was insertionally inactivated using TargeTron technology [27]. Primers were designed using the Sigma Aldrich website (http://www.sigma-genosys.com/targetron/) to allow for re-targeting of the 1.8 kb group II intron, using the vector pJIR3562 [27], to a site 210 nucleotides downstream of the *ccp* start codon, thereby disrupting the gene. Potential mutants were selected based on the presence of an active *erm*(B) erythromycin resistance cassette located within the integrated group II intron and then screened for the absence of zones of precipitation on skim milk agar. Two independent *ccp* mutants were isolated and their genotype confirmed by Southern hybridisation. The results showed that a *ccp-*specific probe hybridised with a 3.9 kb *Hin*dIII fragment in DNA from the wild-type strain and a 5.7 kb fragment in the mutants, as expected since these derivatives contained a 1.8 kb insertion within the *ccp* gene (data not shown). The 5.7 kb fragment also hybridised with an *erm*(B)-specific probe. The plasmid pJIR3680, which contains the full-length *ccp* gene with its natural promoter, was used to complement the *ccp* mutation.

**Figure 1:**
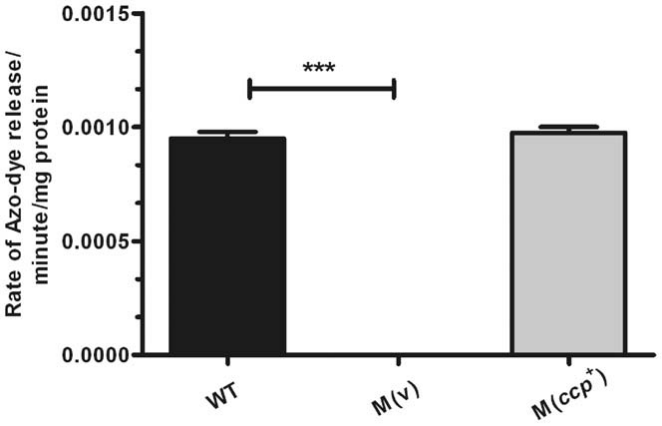
The *ccp* mutant has no detectable protease activity. Culture supernatants (n = 4) isolated at 3.5 h from the wild-type strain JIR325 (WT), a *ccp* mutant carrying the vector plasmid pJIR750 M(v) (JIR12503), and the *ccp* mutant carrying the *ccp^+^* complementation plasmid pJIR3680, M(*ccp^+^)* (JIR12501) were assayed for protease activity as previously described [26] and results expressed as the rate of azo-dye release/minute/mg protein. Error bars indicate S.E.M (*** *p*<0.0001) using a two-tailed unpaired student's t-test comparing the WT and M(v).

Quantitative protease assays showed that the *ccp* mutant carrying the vector plasmid pJIR750 had no detectible protease activity when compared to the wild-type strain (Fig. 1). Protease activity was restored to wild-type levels when pJIR3680 was used to complement the *ccp* mutation. These data indicate that α-clostripain is the major extracellular protease produced by derivatives of *C. perfringens* strain 13.

### Disruption of *ccp* does not affect α-toxin and perfringolysin O production or hemoglobin degradation

**Figure 2:**
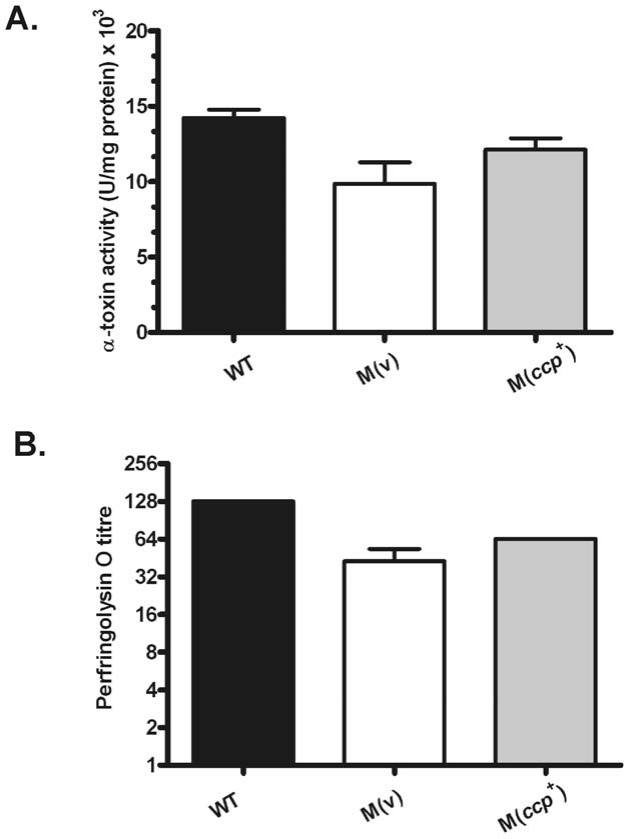
α-toxin and perfringolysin O activity in culture supernatants of isogenic strains. Mid-exponential phase culture supernatants from the wild-type strain (WT), a *ccp* mutant carrying the vector plasmid pJIR750, M(v), and the *ccp* mutant carrying the *ccp^+^* complementation plasmid pJIR3680, M(*ccp^+^*), were either concentrated 5-fold or used undiluted to assay for α-toxin (**A**) or perfringolysin O activity (**B**), respectively (n = 3). Error bars represent S.E.M.

Previous studies have shown that α-clostripain is involved in the processing and degradation of extracellular proteins produced by *C. perfringens* [25]. Therefore, we used quantitative toxin assays to determine if the *ccp* mutation altered the levels of the two major extracellular toxins, α-toxin and perfringolysin O. No significant differences in α-toxin (Fig. 2A) or perfringolysin O activity (Fig. 2B) were observed in supernatants derived from exponential growth phase cultures of wild-type strain, the *ccp* mutant carrying the vector plasmid or the complemented mutant. These results strongly suggest that α-clostripain is not involved in the processing or degradation of either of these two major toxins.

**Figure 3:**
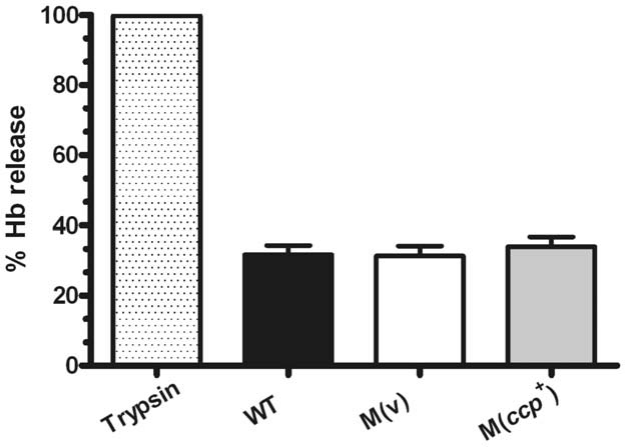
Hydrolysis of hemoglobin. Culture supernatants from mid-exponential phase cultures of the isogenic strains described in Fig. 1 were used to assay for hemoglobin (Hb) hydrolysis. Culture supernatants (n = 4) were mixed with equal volumes of human hemoglobin (1 mg/ml) and incubated for 24 h. Trypsin (0.5%) was used as a positive control. Hemoglobin was precipitated using 3% TCA and the absorbance of the supernatants analysed at 410 nm. The results are shown relative to the amount of hemoglobin hydrolysed by trypsin after 24 h. Error bars indicate S.E.M.

Acquisition of iron from iron-rich proteins like hemoglobin is one of the major factors that influence the progression and establishment of many bacterial infections [28]. The cysteine protease falcipain-3 from *Plasmodium falciparum*, for example, has been shown to degrade human hemoglobin, which is a key mechanism of nutrient acquisition [29], [30]. More recently, the extracellular protease LepA from *Pseudomonas aeruginosa* also was shown to degrade hemoglobin, thereby enabling the bacterium to acquire peptides and heme, and was found to contribute to the growth and virulence of this bacterium, cooperatively with PlcH, a haemolytic phospholipase C [31]. Therefore, we decided to assess the ability of α-clostripain to degrade human hemoglobin, which may be an important nutrient source for *C*. *perfringens* cells in infected host tissues. We developed a heme release assay to measure the cleavage and release of human hemoglobin. Briefly, human hemoglobin was mixed in equal quantities with culture supernatants from the isogenic wild-type, *ccp* mutant and complemented strains. Incubation with trypsin, which was used as a positive control, showed that heme was released under these physiological conditions. By contrast, no difference in the level of hemoglobin degradation was observed between the wild-type, the *ccp* mutant and the complemented strains (Fig. 3), suggesting that α-clostripain is not be involved in hemoglobin degradation under these conditions.

### α-clostripain does not affect the host cell cytotoxicity of culture supernatants

**Figure 4:**
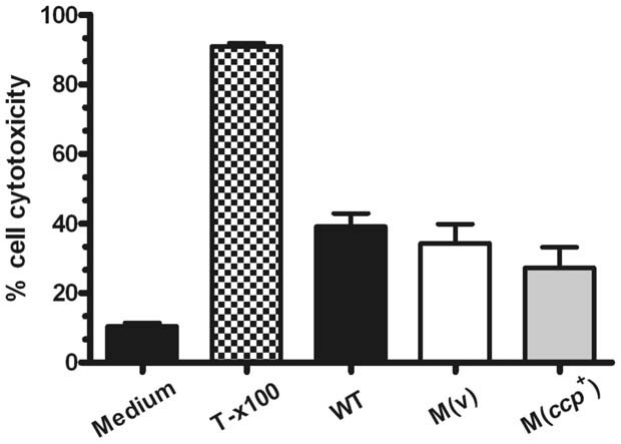
Cytotoxicity of isogenic strains. Differentiated C2C12 cells were stimulated for 24 h at 37°C in 5% CO_2_ with filtered mid-exponential phase culture supernatants (n = 3) from the isogenic strains described in Fig. 1. Cell viability then was assessed using an MTT assay. Culture medium was used as negative control and 1% Triton-X100 (v/v) was used a positive control. Error bars represent S.E.M.

*C. histolyticum* α-clostripain has been shown to induce cell death and cytopathic effects on tissue culture cells, even at low dilutions [32]. Furthermore, purified HrgpA and RgpB, live *P. gingivalis* cells, and filtered culture supernatants were capable of inducing apoptosis in primary human gingivial epithelial cells unlike heat-killed *P. gingivalis* cells [33]. Since previous work suggested that mutation of the α-clostripain gene had the potential to alter the levels of secreted *C. perfringens* proteins [13], [34], and therefore to potentially affect their interaction with host cells, we decided to determine the effect of isogenic culture supernatants on host cell survival. Differentiated DC2C12 mouse muscle cells were incubated with filter-sterilised culture supernatants from the wild-type, *ccp* mutant and complemented strains for 24 h and cellular cytotoxicity determined using an MTT (3-(4,5-dimethylthiazol-2-yl)-2,5-diphenyltetrazolium bromide) cell viability assay (Fig. 4). Treatment with 1% Triton-X100 (v/v) was used a positive control. The results showed that there was no statistically significant difference in DC2C12 cell cytotoxicity upon treatment with these culture supernatants.

### α-clostripain is not essential for virulence in the mouse myonecrosis model

**Figure 5:**
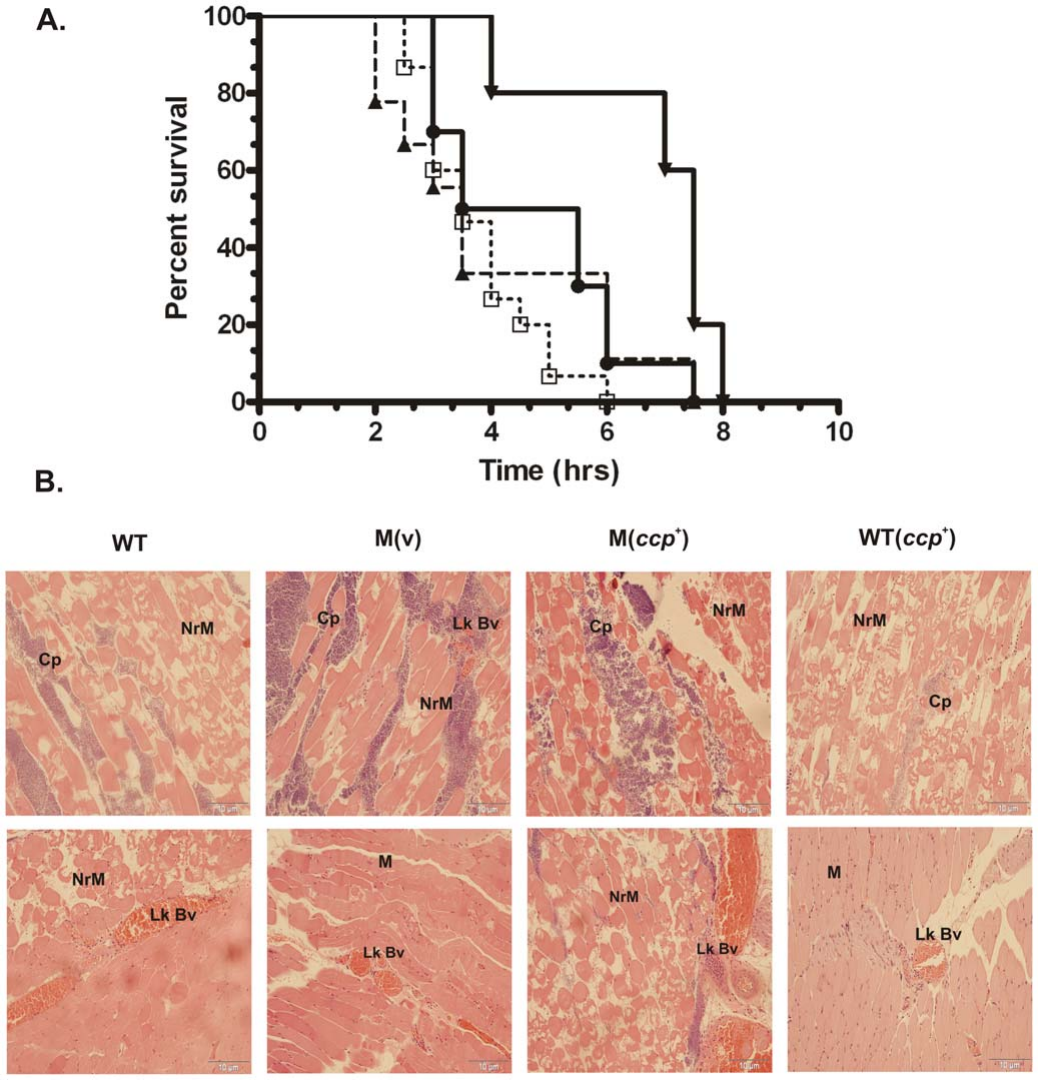
Kaplan-Meier survival curve and histopathology of infected mice. (**A**) Kaplan-Meier survival curve. Female Balb/c mice were inoculated into the right hind thigh muscle with the strains indicated (n = 15). Mice were then observed and scored for signs of disease over 12 h. Legend: • Wild-type (WT), □M(v), ▴M(*ccp^+^*), ▾ Wild-type (*ccp^+^*). (*p*>0.05 using log-rank Mantel-Cox test comparing wild type with mutant and complemented strains) (**B**) Histopathological analysis (hemotoxylin and eosin stain) of muscle tissue isolated from mice infected with the strains indicated. Tissues showed the development of the characteristic signs of disease. **M**-muscle tissue, **Nr M-** necrotic muscle, **Lk Bv-** leukostatic blood vessel, **Cp-** *C. perfringens.*

Finally, the virulence of the isogenic *ccp* strains was assessed in our mouse myonecrosis model. Balb/c mice were injected intramuscularly with live, washed bacteria (10^9^ cfu) [35], [36]. Disease progression was analysed over 12 h and scored for characteristic disease pathology, including blackening of the footpad and the thigh, swelling of the footpad and the thigh and limping, as before [35], [36]. The results showed that mice injected with the *ccp* mutant succumbed to disease at the same rate as those infected with the wild-type or complemented strains (Fig. 5A). A wild-type strain containing the *ccp^+^* complementation vector was also constructed to assess its *in vivo* effects. Virulence testing revealed that although it appeared that disease onset was delayed in this strain, the Kaplan-Meier survival curve was not statistically different from wild type (*p*>0.05, Mantel-Cox test) (Fig. 5A). No differences were seen in the other disease parameters, including blackening of the thigh, footpad or limping, in all of the tested strains (data not shown).

Histopathological analysis of muscle tissues from mice infected with the isogenic panel of strains showed that all of these strains displayed the same pathological characteristics associated with normal *C. perfringens* disease [7], including vascular leukostasis and necrosis of the infected muscle tissues (Fig. 5B). Taken together, the results show that mutation of the α-clostripain gene does not affect the progression or development of clostridial myonecrosis in the mouse model.

## Discussion

The ability of bacterial cells to survive within the host is dependent upon numerous interrelated factors, including the ability of the organism to both evade the host immune response and acquire nutrients that allow its replication and survival. It was previously shown that when purified α-clostripain from *C. perfringens* was injected intradermally into mice there was an increase in vascular permeability [16], thereby implying that α-clostripain may play a role in the pathogenesis of clostridial myonecrosis. This study used only purified α-clostripain and did not consider the concentration of α-clostripain within an infected lesion or its potential interactions with other bacterial and host factors. These findings were in contrast to previous studies, which have shown that an important characteristic of clostridial myonecrosis is an absence of vascular permeability due to the formation of platelet-leucocyte and platelet-platelet aggregates within the vasculature and the up-regulation of adhesion molecules on endothelial cells, leading to an occlusion of blood flow into infected regions. [4], [37]–[40]. Studies using an *in vivo* microvascular perfusion model also have shown that treatment of mouse cremaster muscle tissue with culture supernatants from wild-type *C. perfringens* strains led to a decreased functional capillary density [40], which was reversed when cells were pre-treated with anti-Gr-1, a granulocyte-specific antibody, and anti-platelet serum [40]. These data confirmed the importance of neutrophil-platelet aggregates in blocking vascular perfusion during *C. perfringens* infection.

Using a mouse myonecrosis infection model, we have now shown that α-clostripain is not essential for *C. perfringens*-mediated disease. Mice infected with an α-clostripain mutant did not show any alteration in disease progression or in the development of vascular leukostasis, a hallmark of *C. perfringens* infection (Fig. 5). Mutation of the *ccp* gene also had no affect on bacterial growth or on the extracellular level of the two major toxins, α-toxin and perfringolysin O (Fig. 2), which are implicated in clostridial myonecrosis [1], [7]. In agreement with previous studies [41] our results provide good evidence that α-clostripain is the major protease produced by *C. perfringens* strain 13 (Fig. 1). Other bacterial cysteine proteases, which unlike α-clostripain have been shown to be essential for virulence, also are involved in influencing the levels of other virulence factors, many of which have non-redundant functions. For example, mutation of both *P. gingivalis* gingipains not only reduces total cysteine protease activity, but also affects the growth and expression of cell surface structures such as fimbriae and vesicles, both of which are thought to assist in colonization and evasion of the immune response [42]–[44].

Like α-clostripain other potential *C. perfringens*-encoded spreading factors, such as collagenase and two different sialidases, are not to be essential for disease in the mouse myonecrosis model [3], [45]. These enzymes may still have a role during infection. The breakdown of host tissues is important for the release of nutrients such as amino acids, sugars and essential minerals such as sequestered iron, which are required for the growth of *C. perfringens* cells in the lesion. We previously postulated that the sialidases secreted by *C. perfringens* may still serve such a function even though they are not essential for virulence [45]. Accordingly, we tested the ability of α-clostripain to hydrolyse human hemoglobin since other bacterial proteases can catalyze the release of heme from hemoglobin. Although some hemoglobin degradation was observed, there was no significant difference between the wild-type strain and the *ccp* mutant, suggesting that α-clostripain does not play a major role in hemoglobin hydrolysis.

The clostridial mouse myonecrosis model used in this study, and many other studies [1], [3], [7], [45], is the only animal model currently available that allows the consistent reproduction of virulent disease. However, since the model involves injection of anaerobic bacteria into healthy oxygenated tissue, an infectious dose of 109 cells is required to establish reproducible disease. This process may mask any role that extracellular enzymes such as α-clostripain, sialidase or collagenase may have during the early stages of the disease process. Therefore, we cannot rule out the possibility that α-clostripain has a role in disease pathogenesis in a natural *C. perfringens* infection, where a traumatic injury usually leads to the establishment of ischemic conditions, enabling the proliferation of small numbers of contaminating cells in the muscle tissues leading to a major infection and fulminant gas gangrene.

## Materials and Methods

### Ethics Statement

Animal experiments were approved by the Monash University School of Biomedical Sciences B (SOBSB) Animal Ethics Committee (Approval number: SOBSB/M/2009/16) and were conducted in accordance with Victorian State Government regulations. All efforts were made to ameliorate any unnecessary suffering of animals.

### Bacterial strains and growth conditions

All *C. perfringens* strains were derivatives of JIR325, a rifampicin and nalidixic acid resistant derivative of strain 13 [46]. *C. perfringens* strains were grown in tryptone-peptone-yeast extract glucose (TPYG) broth supplemented with 0.375% glucose (v/v) and 0.1% sodium thioglycolate (w/v) [47], fluid thioglycolate broth (FTG) (Difco), or nutrient agar (2.5% Nutrient Broth (Oxoid), 0.3% yeast extract (Oxoid), 0.1% sodium thioglycolate, 1.5% agar (Oxoid)), supplemented when necessary with 10 µg/ml rifampicin and 10 µg/ml nalidixic acid for wild-type *C. perfringens* strains, 10 µg/ml erythromycin for α-clostripain mutants or 30 µg/ml chloramphenicol for complemented strains. Skim milk agar was made by adding 2% skim milk powder to nutrient agar. Broth cultures were boiled for 5–10 min prior to inoculation to render them anaerobic and agar cultures were grown at 37°C in 10% (v/v) H2, 10% (v/v) CO2 in N2.

### Molecular and genetic techniques

*C. perfringens* genomic [48] and plasmid DNA [49], and *E. coli* plasmid DNA [50], [51] were isolated as previously described. Plasmid DNA was introduced into *C. perfringens* by electroporation at 1.8 kV, 25 µF and 200 Ω [52]. TargeTron technology was used to mutate the α-clostripain gene as previously described [27], using a retargeted derivative of pJIR3562 [27]. Target sites were chosen based on the best score provided on the Sigma Aldrich website (http://www.sigma-genosys.com/targetron/) and primers were designed to allow retargeting of the group II intron between nucleotides 210 and 211 on the sense strand, at the start of the *ccp* gene. *C. perfringens* transformants were selected on erythromycin-containing media and were screened for the absence of proteolytic activity on skim milk agar as well as for thiamphenicol sensitivity. The resultant α-clostripain mutant was designated as JIR12337 and, when transformed with the plasmid vector pJIR750, as JIR12503.

### Complementation of the *ccp* gene

The wild-type *ccp* gene, including 200 base pairs upstream of the start site to incorporate its putative promoter, were subcloned into pCR-Blunt II TOPO (Invitrogen) to construct pJIR3679. This plasmid and the *E. coli* - *C. perfringens* shuttle plasmid pJIR750 [53], which carries the *catP* chloramphenicol resistance gene, were then digested with *Bam*HI (Roche) and the digested fragments mixed and ligated with high concentration T4 DNA ligase (Roche). The ligated mixture then was used to transform chemically competent *E. coli* DH5α cells [51] and recombinants selected based on their chloramphenicol resistance and proteolytic activity on skim milk agar. The complementation plasmid, pJIR3680, was confirmed by restriction analysis and sequencing and used to transform the *C. perfringens ccp* mutant JIR12337 to chloramphenicol resistance [52]. Colonies were selected on skim milk nutrient agar supplemented with 30 µg/ml chloramphenicol and screened for the restoration of zones of proteolysis. The complemented strain was designated as JIR12501. A wild-type strain containing the *ccp* complementation vector was also constructed by electroporation of JIR325 with pJIR3680 and was designated as JIR12539.

### Toxin and enzyme assays

Relevant strains were grown to mid-exponential phase (3.5 h) in 100 ml of TPYG broth; the strains all grew at the same rate. Cells were removed by centrifugation at 8,500 *g* for 10 min at room temperature. α-toxin assays were performed as previously described, with activity presented as units/mg total protein [54] and total protein determined using a BCA assay kit (Pierce). Perfringolysin O activity was determined using a doubling-dilution assay as previously described [27], [55] with the addition of 5 mM dithiothreitol (DTT) (Roche). The haemolytic titre was expressed as the highest dilution that caused at least 80% haemolysis. Protease assays were performed as previously described [26]. Briefly, culture supernatants were mixed with 5 mg/ml azocasein (Sigma Aldrich) dissolved in 25 mM Tris-HCl buffer, pH 7.5, supplemented with 5 mM DTT and incubated for 2 h at 37°C with gentle rotation. The proteins were then precipitated from solution with the addition of 3% (w/v) TCA and azocasein hydrolysis determined by reading the absorbance of the supernatants at 570 nm. Protease activity was defined as the rate of azo-dye release/per minute/mg of protein.

### Hemoglobin degradation assay

Culture supernatants were mixed in equal quantities with 1 mg/ml human hemoglobin (Sigma Aldrich) in 25 mM Tris-HCl buffer (pH 7.5), supplemented with 5 mM DTT and incubated for 24 h at 37°C. Trypsin (0.5%) (Invitrogen) was used a positive control and medium only was used as a negative control. After incubation, the mixtures were precipitated with 3% (w/v) trichloroacetic acid (TCA) and heme release determined by measuring the absorbance of the supernatants at 410 nm.

### Cell cytotoxicity assays

The mouse muscle cell line C2C12, was maintained in Dulbecco's Modified Eagles Medium (DMEM) (Invitrogen) supplemented with 10 mM L-glutamine and 10% (v/v) heat inactivated foetal bovine serum (FBS) (Sigma Aldrich). To obtain differentiated C2C12 cells (DC2C12 cells) for subsequent experiments, C2C12 cells (ATCC) were allowed to grow to 80 to 90% confluence and then aliquoted into 96 well microtitre plates at 2×10^4^ cells well (2×10^5^ cells/mL) in DMEM with 2% FBS and incubated for a further five days. The differentiated cell monolayers then were stimulated with filter sterilised (0.4 µm) culture supernatants from 3.5 h *C. perfringens* cultures for 24 h at 37°C in 5% CO2. Culture medium was used as a no stimulation control and treatment with 1% (v/v) Triton-X100 was used as a total cell lysis control. Cellular cytotoxicity was measured using an MTT assay (Biocore) as per the manufacturer's instructions. Percentage cell death was calculated after subtracting the absorbance from cells stimulated with medium only from the absorbance of treated cells.

### Virulence studies

Virulence trials were conducted as described previously [6], [7]. Briefly, *C. perfringens* cultures were grown for 6 h in FTG broth and then subcultured onto nutrient agar with appropriate antibiotic selection. After overnight growth the cells were removed into Dulbecco's phosphate buffered saline (DPBS), washed in DPBS and resuspended in three times the cell pellet volume in DPBS. Female Balb/c mice were injected into the right hind thigh muscle with 50 µl of this suspension (approximately 10^9^ cfu) and monitored every 30 min for 12 h for the characteristic signs of disease pathology: blackening of the thigh and footpad, swelling of the thigh and footpad and limping [6], [7], [56]. Mice were euthanised when they developed significant disease pathology. Viable counts of each *C. perfringens* stain were performed both pre- and post-infection. Histopathology was performed on infected thigh tissue collected from mice immediately following euthanasia and stored in 10% phosphate buffered formalin. Tissues were sliced into 5 µm sections and stained with haematoxylin and eosin by Monash University Histology Services. Stained sections were analysed using an Olympus BX51 microscope with a DP70 camera.
